# Micheliolide suppresses LPS-induced neuroinflammatory responses

**DOI:** 10.1371/journal.pone.0186592

**Published:** 2017-10-17

**Authors:** Zhaomeng Sun, Guodong Li, Tanjun Tong, Jun Chen

**Affiliations:** 1 Peking University Research Center on Aging, Beijing, China; 2 Department of Biochemistry and Molecular Biology Peking University Health Science Center, Beijing, China; National Institutes of Health, UNITED STATES

## Abstract

Microglia-involved neuroinflammation is thought to promote brain damage in various neurodegenerative disorders. Thus, inhibition of microglial over-activation may have a therapeutic benefit for the treatment of neurodegenerative disorders. Micheliolide (MCL) is a sesquiterpene lactone which inhibits various inflammatory response. However, whether MCL can inhibit neuroinflammation caused by LPS-activated BV2 microglia has not yet been explored. In this study, we demonstrated that treatment of BV2 cells with MCL significantly repressed LPS-stimulated nitric oxide synthase (iNOS) and cyclooxygenase-2 (COX-2) expression, as well as tumor necrosis factor-alpha (TNF-α), interleukin-6 (IL-6) and nitric oxide (NO) induction. MCL also attenuated mRNA levels of multiple pro-inflammatory cytokines and mediators such as iNOS, COX-2, TNF-α, IL-6 and IL-1β. Mechanistic studies revealed that MCL suppressed LPS-stimulated the activation of IκBα/NF-κB pathway and Akt pathway. Moreover, MCL inhibited LPS-induced the activition of c-Jun N-terminal kinase (JNK), p38 MAPK kinase, and extracellular signal-regulated kinases 1/2 (ERK1/2). Meanwhile, MCL markedly promoted antioxidant protein heme oxygenase-1 (HO-1) expression by enhancing NF-E2-related factor 2 (Nrf2) activity. Together, our results imply that MCL may serve as a neuroprotective agent in neuroinflammation-related neurodegenerative disorders.

## Introduction

Microglia, resident macrophages in the central nervous system (CNS), are crucial players of the innate immune responses and serve as the frontline of defense against foreign substances and pro-inflammatory response [1, 2]. In the homeostatic state, microglia function in the host protection of brain, and act as phagocytes to clean up damaged neurons and tissue debris [2, 3]. However, aberrantly activated microglia significantly increase neuroinflammation and neurotoxicity by secreting various pro-inflammatory cytokines and mediators including TNF-α, interleukin-1β (IL-1β), interleukin-6 (IL-6), NO, reactive oxygen species (ROS), inducible nitric oxide synthase (iNOS), and COX-2 etc., which can lead to neurodegenerative diseases such as Parkinson’s disease (PD), Alzheimer’s disease (AD), cerebral ischemia, multiple sclerosis, and stroke [4–8]. Therefore, the candidate drugs that target the aberrant activation of microglia may have valuable therapeutic potential for the treatment of neuroinflammation-related diseases.

Multiple signaling pathways are implicated in modulating microglial activation. NF-κB is a predominant transcription factor in regulating pro-inflammatory mediators [9]. Inhibition of NF-κB activity is widely recognized as a good strategy for suppressing neuroinflammation. In addition, mitogen-activated protein kinase (MAPK) signaling cascades including c-Jun N-terminal kinase (JNK), p38, and extracellular signal-regulated kinase ERK1/2 also modulate microglial inflammatory responses through activating NF-κB thereby enhancing cytokine expression [3, 10, 11]. Recent studies demonstrate that the phosphatidyl inositol 3-kinase/Akt (PI3K/Akt) pathway is essential for efficient activation of NF-κB and subsequent inflammatory genes expression [12]. The transcription factor NF-E2-related factor 2 (Nrf2)/antioxidant response element (ARE) signaling pathways are thought to be the central modulator of anti-inflammation and neuroprotection [13, 14]. Nrf2 regulates the transcription of antioxidant genes including heme oxygenase-1 (HO-1) and NAD(P)H:quinone oxidoreductase 1 (NQO-1) [11]. HO-1 has been suggested as a potential therapeutic target for treating many neuroinflammatory diseases [15].

Micheliolide (MCL) is a guaianolide sesquiterpene lactone isolated from Michelia compressa and Michelia champaca [16]. MCL can cross the blood-brain barrier (BBB), a formidable obstacle for drugs to exert a therapeutic effect in vivo, and preferentially accumulates in the brain [17]. To date, research on MCL mainly focuses on the antitumor activity, such as acute myelogenous leukemia (AML) [18–21], malignant gliomas [17] and breast cancer [22, 23]. Recently, MCL has also been described to possess anti-inflammatory properties. For example, MCL has been reported to suppress LPS-induced inflammatory response and protects mice from LPS challenge via inhibition of NF-κB and PI3K/Akt activities [24]. Moreover, MCL is reported to inhibit dextran sodium sulphate (DSS)-induced inflammatory intestinal disease, colitis-associated cancer and rheumatic arthritis [25, 26]. Despite of the anti-inflammatory potentials of MCL showing in these studies, whether MCL can suppress microglial overactivation-caused neuroinflammation which induced by LPS challenge is largely unknown. In this study, we investigated the anti-inflammatory effect of MCL on LPS-stimulated neuroinflammation in vitro and in vivo.

## Results

### MCL treatment did not induce cytotoxicity in BV2 cells

**Fig 1:**
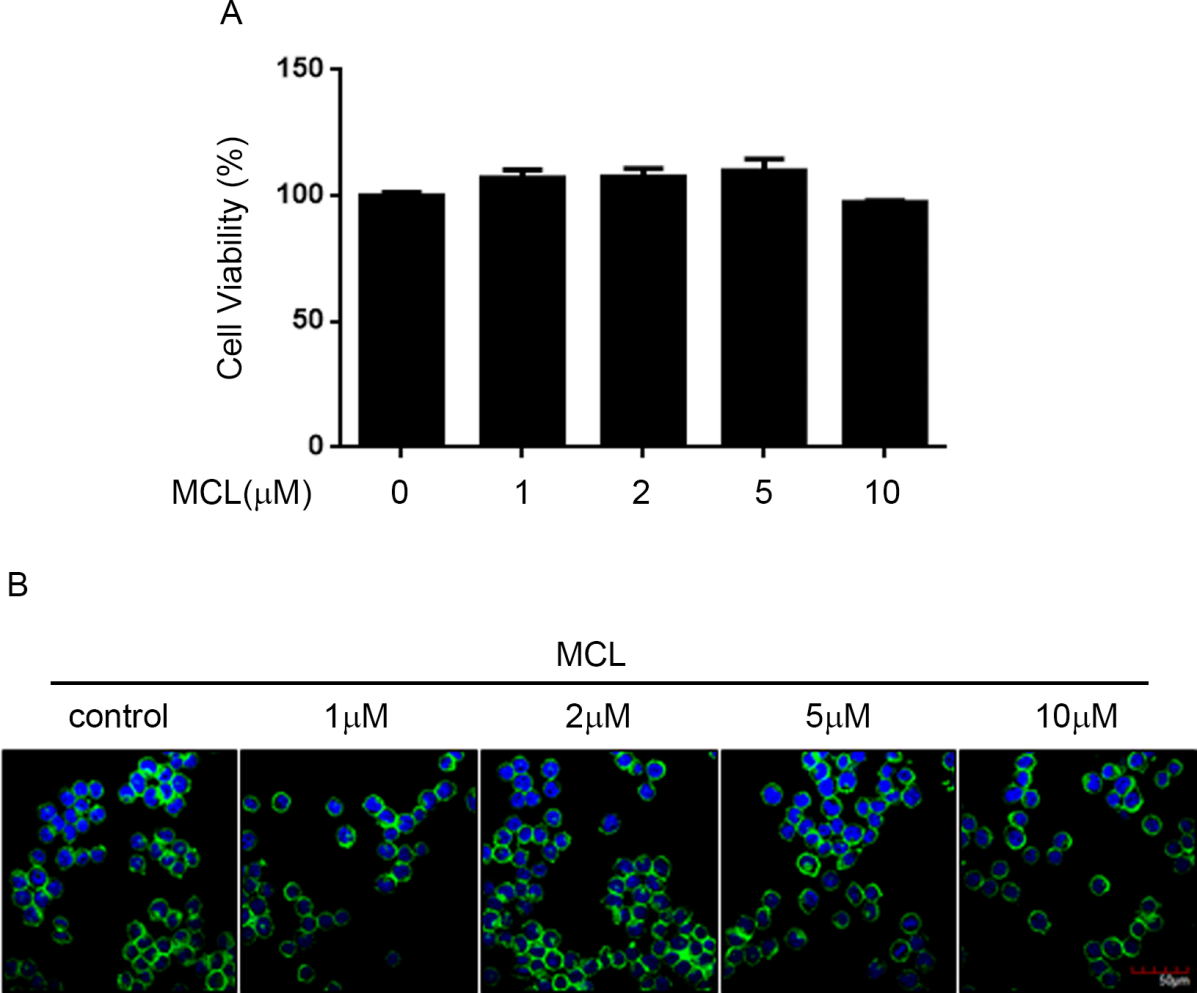
MCL treatment did not affect BV2 cell viability. (A) BV2 cells were treated with the indicated doses of MCL for 24 h, then the cytotoxicity of MCL was measured by CCK-8 assay. The cell viability result was normalized to BV2 cells without MCL treatment for each other. Data were presented as means ± SD of three independent experiments. (B) BV2 cells were treated with indicated doses of MCL for 24 h. Then, the cells were stained with FITC-Phalloidin, green; and nuclei, Hoechst33258, blue; representative images by immunofluorescence were shown, scale bar = 50 μm.

Prior to investigation the effects of MCL on BV2 cells, CCK-8 assay was performed to determine its cytotoxicity to BV2 cells. After 24 hours incubation with different concentrations of MCL, cell viability of BV2 cells was not significantly altered by any doses of MCL treatment from 1 μM up to 10 μM (Fig 1A). To further evaluate whether MCL treatment induce BV2 cytotoxicity, we used phalloidin and Hoechst to double stain the cells. The immunofluorescent result showed that none of the dosage of MCL induced any alteration in BV2 cell morphology (Fig 1B). These results indicated that the dosage of MCL used in this study did not trigger BV2 cytotoxicity.

### MCL inhibited LPS-induced iNOS, NO and COX-2 expression in BV2 cells

**Fig 2:**
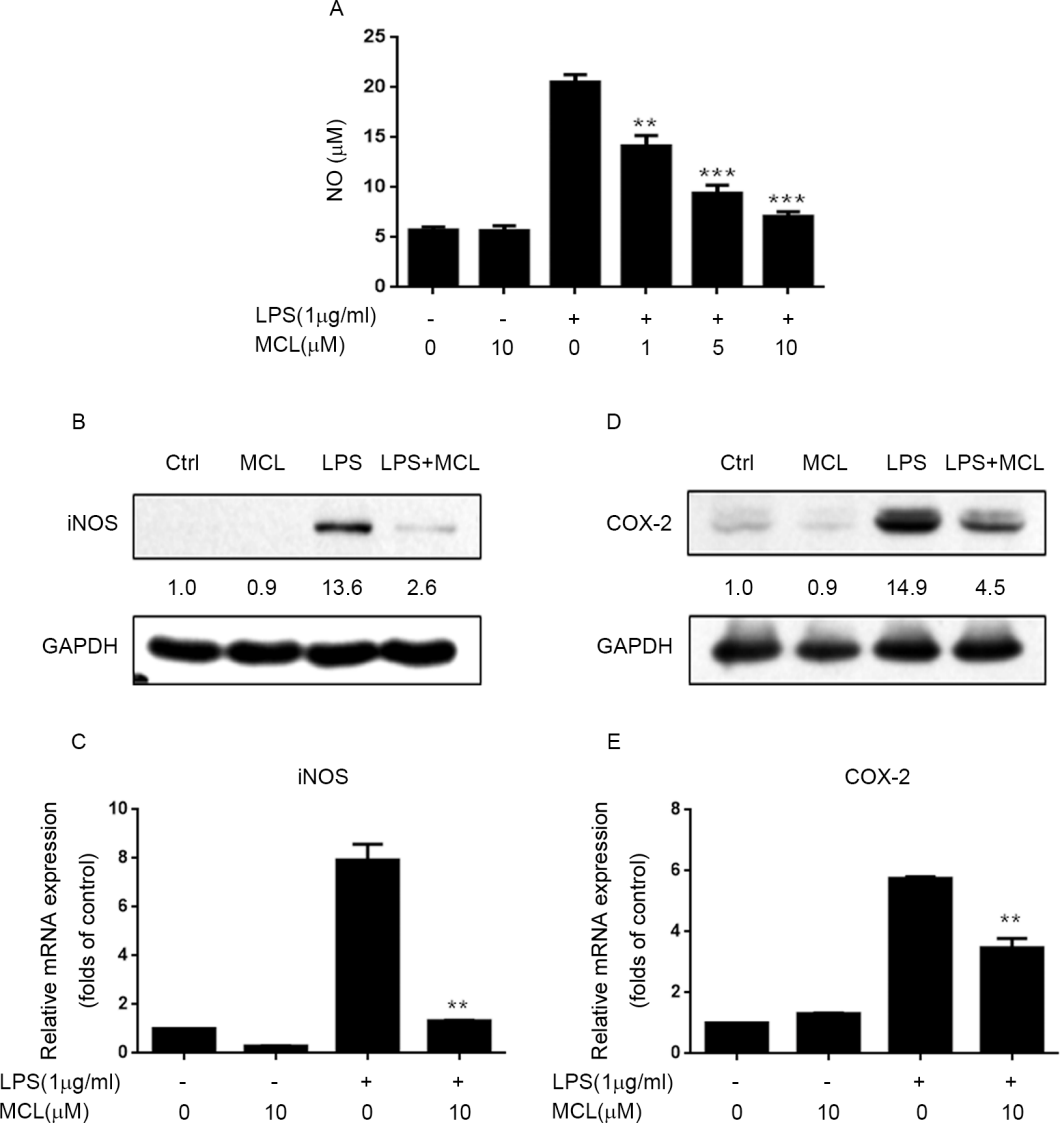
MCL inhibited LPS-induced iNOS and COX-2 expression, and NO production in BV2 cells. (A) BV2 cells were pretreated with MCL (1, 5, and 10 μM) for 1 h and then incubated with LPS (1 μg/ml) for 24 h. The NO concentration in supernatant was determined using Griess reagent. (B) In a parallel experiment, cell lysates were subjected to Western blot analysis and immunoblotted with the indicated proteins. (C) BV2 cells were pretreated with MCL (10 μM) for 1 h and then incubated with LPS (1 μg/ml) for 6 h. Then, mRNA was extracted and the mRNA level of iNOS was evaluated by RT-PCR. (D) BV2 cells were pretreated with MCL (10 μM) for 1 h and then incubated with LPS (1 μg/ml) for 24 h. The indicated proteins were evaluated by Western blot. (E) BV2 cells were pretreated with MCL (10 μM) for 1 h and then incubated with LPS (1 μg/ml) for 6 h. Then, mRNA was extracted and the mRNA level of COX-2 was evaluated by RT-PCR. Data were presented as means ± SD of three independent experiments. **p < 0.01 and ***p < 0.001 vs. LPS alone.

First, we examined whether MCL treatment could suppress LPS-stimulated iNOS and NO production in BV2 cells. BV2 cells were pretreated with MCL and followed by LPS challenge, then the NO level in the culture media was determined by Griess assay. (The time lines which shown when the cells were treated with MCL and LPS and collected for any experiments were presented in S1 Fig). In LPS-stimulated BV2 cells, there was a marked increase in NO production compared to unstimulated cells. In contrast, MCL pretreatment significantly decreased NO production in a dose-dependent manner (Fig 2A). It is well known that NO production mainly relies on the iNOS expression. We then assessed whether MCL reduced NO level via suppression of iNOS induction. As shown in Fig 2B and 2C, LPS stimulation significantly promoted iNOS expression at both protein level and mRNA level. However, MCL treatment effectively hampered LPS-stimulated iNOS expression both at protein and mRNA levels (Fig 2B and 2C). These data indicated that MCL restrained LPS-induced NO production in BV2 cells via down-regulation of iNOS mRNA and protein expression.

We further determined whether MCL treatment could inhibit LPS-stimulated COX-2 expression. Similar to iNOS results, MCL significantly impeded COX-2 protein expression (Fig 2D) and mRNA transcription (Fig 2E) upon LPS treatment. These results suggested that MCL could repress LPS-induced COX-2 expression.

### MCL mitigated LPS-induced pro-inflammatory cytokines production in BV2 cells

**Fig 3:**
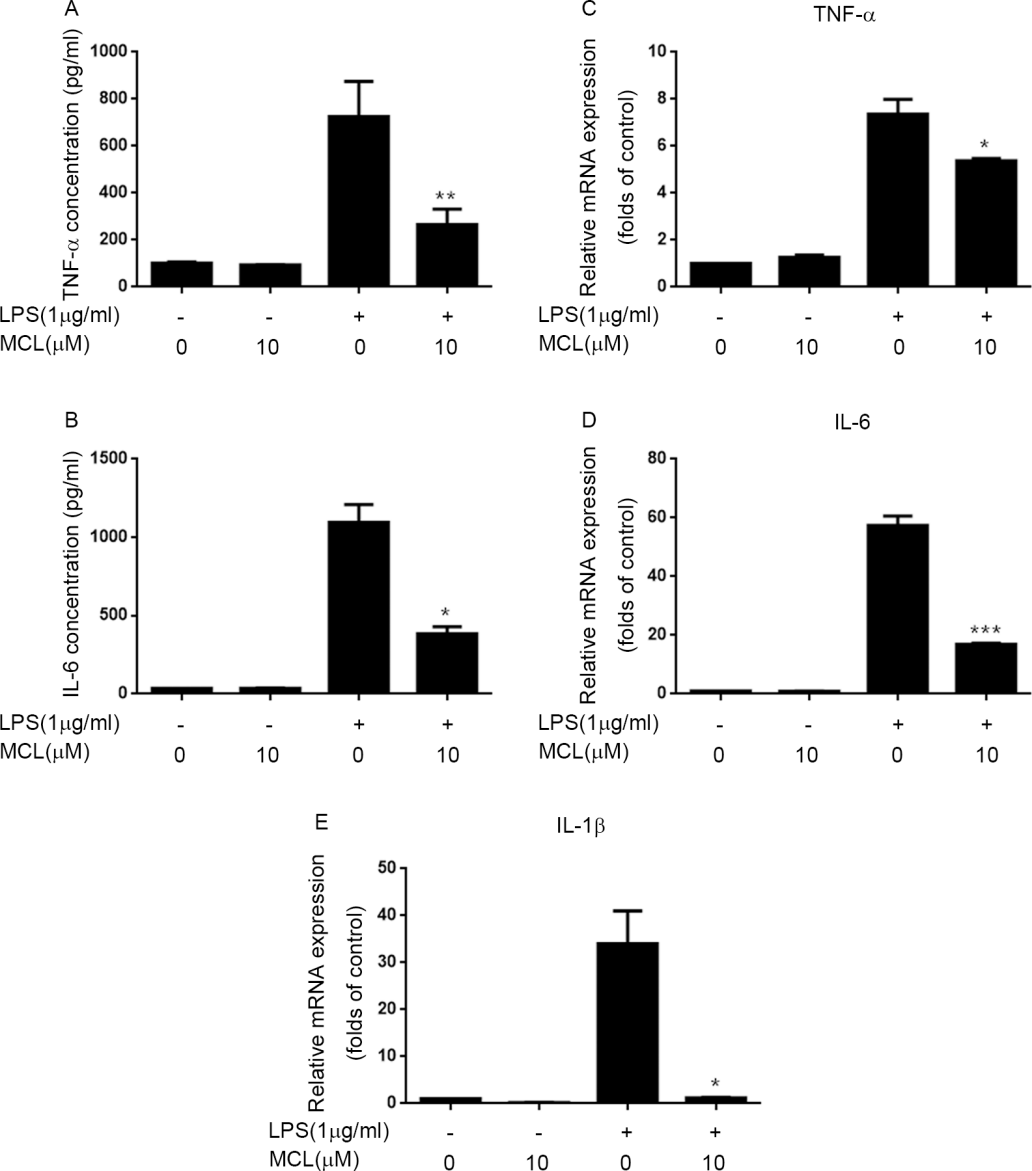
MCL decreased LPS-induced pro-inflammatory cytokines production. (A) and (B) BV2 cells were pretreated with MCL (10 μM) for 1 h and then incubated with LPS (1 μg/ml) for 12 h. The secretory levels of TNF-α and IL-6 in supernatants were measured using ELISA. (C), (D) and (E) BV2 cells were treated with MCL (10 μM) for 1 h and followed by LPS treatment for 6 h. Then, mRNA was extracted and the mRNA level of TNF-α (C), IL-6 (D) and IL-1β(E) was evaluated by RT-PCR. Data were presented as means ± SD of three independent experiments. *p < 0.05, **p < 0.01 and ***p < 0.001 vs. LPS alone.

LPS challenge in BV2 cells induces various pro-inflammatory cytokines production including TNF-α, IL-6 and IL-1β, etc [4–8]. Thus, we determined whether MCL could hinder LPS-induced pro-inflammatory cytokines production. In LPS- stimulated BV2 cells, the secretory levels of TNF-α and IL-6 in supernatant detected by enzyme-linked immunoadsorbent assay (ELISA) were significantly increased compared to unstimulated cells (Fig 3A and 3B). Conversely, pretreatment with MCL substantially attenuated TNF-αand IL-6 secretory levels compared to LPS treatment only (Fig 3A and 3B). We further examined whether MCL could affect mRNA levels of TNF-αand IL-6 by using RT-PCR. Consistent with secretion results, the increment of TNF-αand IL-6 mRNA levels which induced by LPS challenge was significantly alleviated by MCL treatment (Fig 3C and 3D). In addition, we also observed that LPS-stimulated the up-regulation of IL-1βmRNA level was largely diminished by MCL treatment (Fig 3E). These data revealed that MCL could inhibit pro-inflammatory cytokines expression induced by LPS in BV2 cells.

### MCL suppressed LPS-induced NF-κB activity

**Fig 4:**
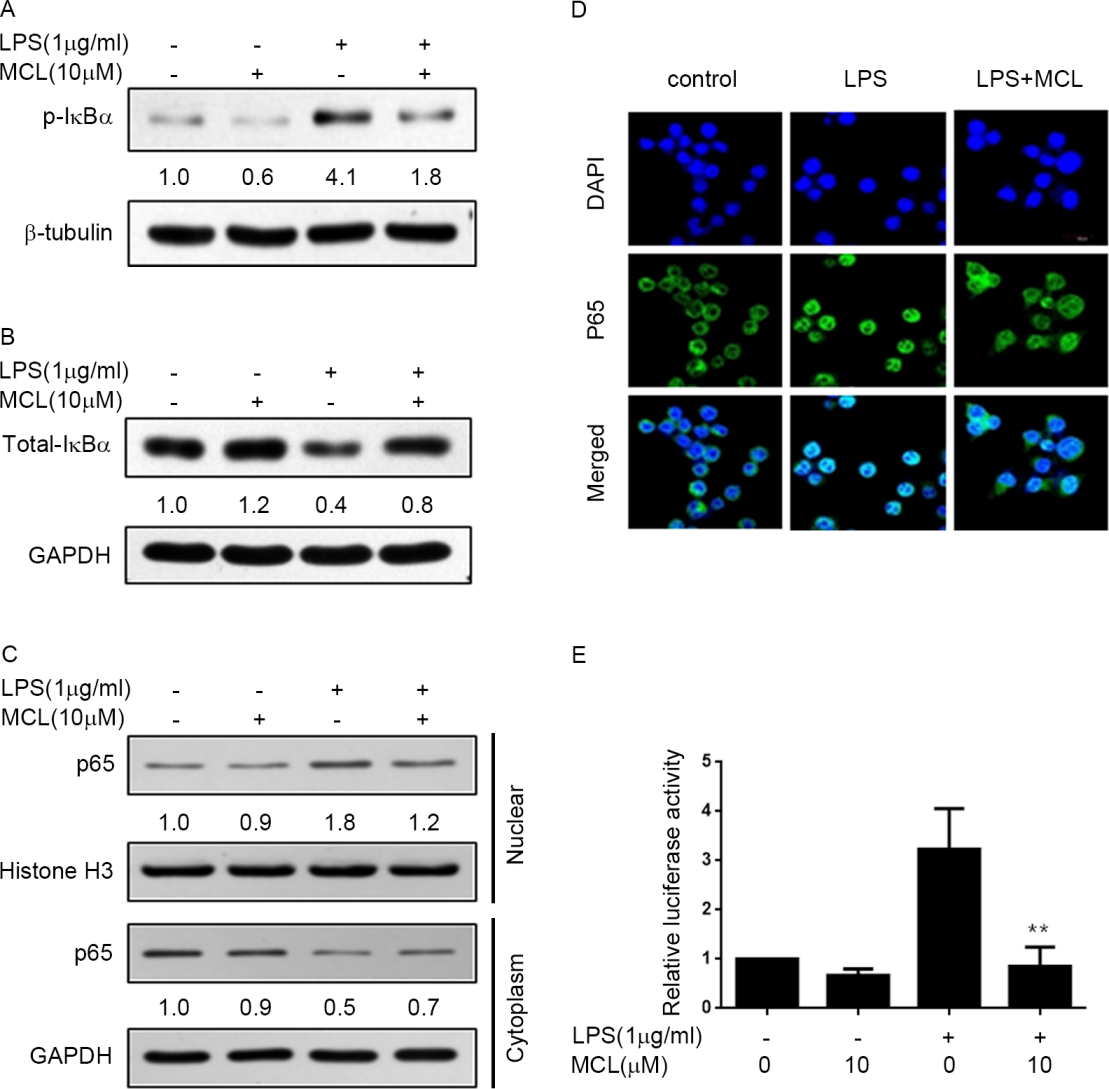
MCL repressed LPS-induced NF-κB activity. (A) and (B) BV2 cells were pretreated with MCL (10 μM) for 1 h and then added LPS (1 μg/ml) for 30 min. Total cell lysates were subjected to Western blot analysis using antibodies against phospho-IκBα (A) or total form of IκBα (B). (C) Cells were treated similar to (A). The cytosolic and nuclear extracts were subjected to Western blot analysis for the indicated proteins. (D) BV2 cells were treated with LPS (1 μg/ml) for 1 h after pretreatment with MCL (10 μM) for 1 h. Then, cells were stained with NF-κB subunit p65 antibody, green; and nuclei, DAPI, blue; scale bar = 20 μm, representative images by immunofluorescence were shown. (E) NF-κB luciferase reporter vector was co-transfected with Renilla vector in BV2 cells. 24 h later, cells were treated with MCL (10 μM) for 1h then followed by LPS (1 μg/ml) incubation for 6 h. Then, cell lysates were collected, and NF-κB luciferase activities against Renilla luciferase activities were measured by the double-luciferase assay system. Data were presented as means ± SD of three independent experiments. **p < 0.01 vs. LPS alone.

It is well known that many pro-inflammatory cytokines induction mainly depends on the NF-κB activity. Thus, we next investigated whether the inhibitory effect of MCL on pro-inflammatory cytokines expression was via suppression of NF-κB activity in BV2 cells. In canonical NF-κB activation pathway, a cytoplasmic IKKα-IKKβ-nemo complex is phosphorylated, thereby causing IκBα phosphorylation and degradation and subsequent NF-κB nuclear translocation. Therefore, we first examined the influence of MCL on the IκBα phosphorylation and degradation. Stimulation of BV2 cells with LPS resulted in the marked increase of phospho-IκBα level and decrease of total IκBα, which was considerably reversed by MCL treatment (Fig 4A and 4B). Next, we investigated whether MCL treatment could block the NF-κB subunit p65 translocation from the cytosol to the nucleus by isolation of nuclear and cytosolic organelles. As shown in Fig 4C, after LPS treatment, the cytosolic p65 level reduced and nuclear p65 level increased when compared to the untreated cells, which indicated that p65 translocated from cytosol to nucleus and NF-κB was activated. Histone H3 signal proved the purity of the nuclear preparations. In contrast, the cytosolic and nuclear p65 levels in MCL-treated cells were similar to unstimulated cells, which indicated that MCL treatment blocked p65 translocation from cytosol to nucleus. The immunofluorescent analysis further demonstrated that LPS induced p65 translocation from cytosol to the nucleus was prevented by MCL treatment (Fig 4D). Furthermore, we determined the effect of MCL on LPS-induced NF-κB promoter binding activity by luciferase reporter gene assay. Our result showed that pretreatment with MCL significantly inhibited LPS-stimulated NF-κB luciferase activity (Fig 4E). These findings implied that MCL hindered LPS-stimulated pro-inflammatory cytokines expression in BV2 cells at least partly via inhibition of IκBα/NF-κB pathway.

### MCL inhibited LPS-induced MAPKs and PI3K/Akt pathways activities

**Fig 5:**
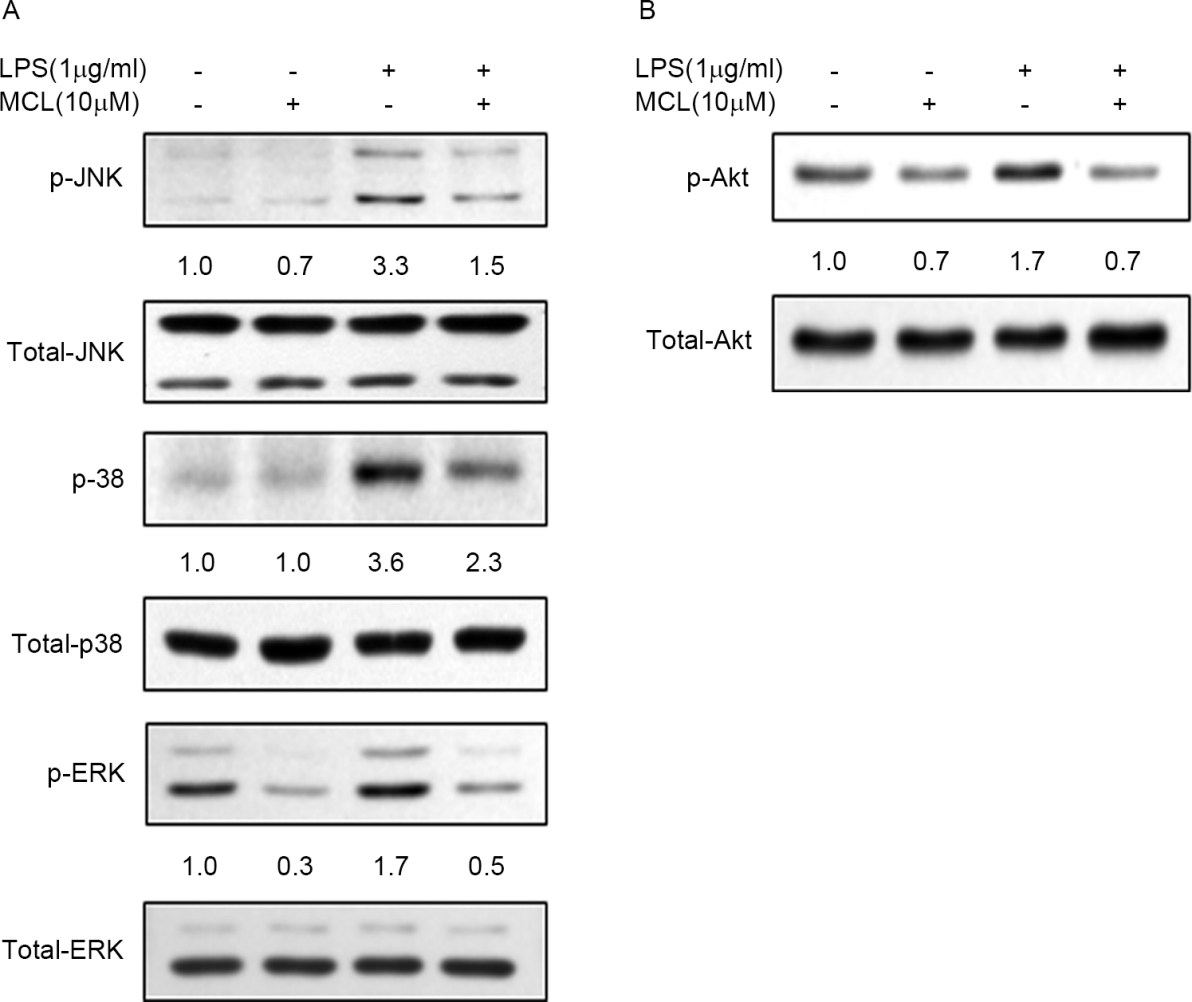
MCL suppressed LPS-induced MAPKs and Akt activities. (A) and (B) BV2 cells were treated with MCL (10 μM) for 1 h then followed by LPS (1 μg/ml) addition for 30 min. Then, total cell lysates were subjected to Western blot analysis using antibodies against phospho- or total forms of JNK, p38, ERK, and Akt.

Previous studies have demonstrated that MAPKs signaling pathway modulate LPS-induced pro-inflammatory cytokines expression through activating NF-κB in microglia [3, 10, 11]. Thus, we sought to explore the effect of MCL on MAPKs pathway. As shown in Fig 5A, the phosphorylation levels of MAPKs including JNK, p38, and ERK significantly elevated in LPS-treated BV2 cells compared to untreated cells, without altering the basal expression level of these MAPKs. In contrast, MCL treatment markedly impeded these MAPKs activities. This observation indicated that MCL might inhibit LPS-stimulated NF-κB activity and pro-inflammatory cytokines expression partially via inhibition of MAPKs signaling pathway.

Since PI3K/Akt signaling pathway is also reported to regulate NF-κB activity and inflammatory response [12], we then detected the effect of MCL on Akt activity. LPS stimulation triggered the significant up-regulation of phosphorylated Akt compared to untreated cells, without affecting the basal level of Akt (Fig 5B). Conversely, MCL treatment remarkably blocked LPS-induced Akt activity. Our result indicated that MCL might also suppress LPS-stimulated NF-κB activity partly through decreasing Akt signaling activity.

### MCL enhanced Nrf2 and HO-1 expression

**Fig 6:**
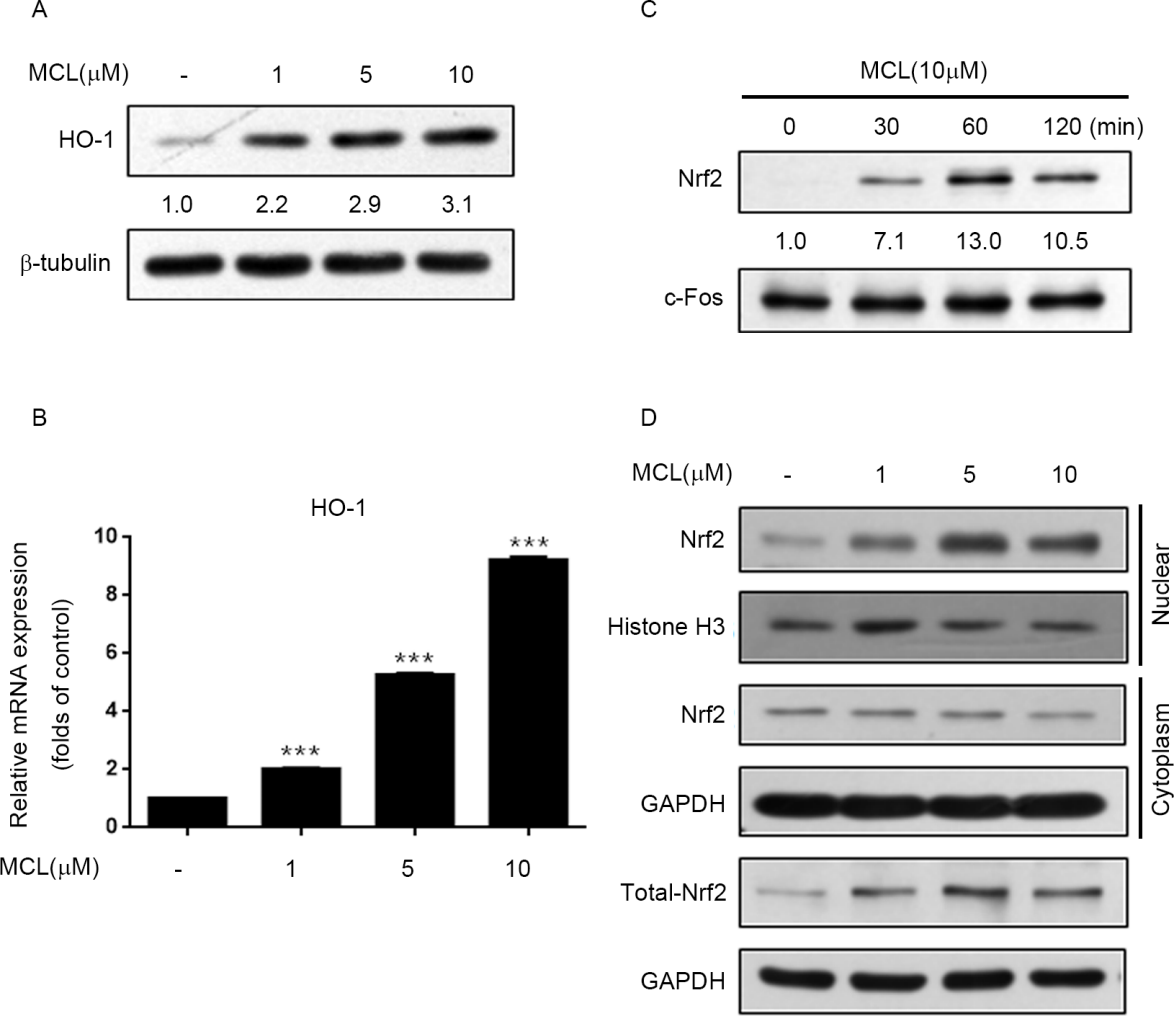
MCL promoted Nrf2 and HO-1 expression. (A) and (B) BV2 cells were treated with the indicated doses of MCL for 12 h. Then, total cell lysates were subjected to Western blot analysis using antibody against HO-1 (A); or total RNA was extracted and the mRNA level of HO-1 was evaluated by RT-PCR (B). (C) and (D) BV2 cells were treated with MCL (10 μM) for the indicated times (C) or treated with various doses of MCL for 2 h (D). Nuclear and cytosolic fractions were isolated and subjected to Western blot analysis using antibody against Nrf2. Data were presented as means ± SD of three independent experiments. ***p < 0.001 vs. control group.

The transcription factor Nrf2 and its target gene HO-1 are thought to execute anti-inflammation and neuroprotection effect on microglia. Therefore, we further analyzed whether Nrf2/HO-1 pathway contributed to the anti-inflammatory effect of MCL on LPS-induced microglial inflammation. As shown in Fig 6A and 6B, MCL treatment promoted HO-1 expression at both protein and mRNA levels in a dose-dependent manner. We further exploited the effect of MCL on Nrf2 nuclear translocation which related to its transcriptional activity. The nuclear accumulation of Nrf2 was increased in MCL-treated cells compared to untreated cells, with the maximum effect being observed at 1 h MCL treatment (Fig 6C). Moreover, we found that MCL treatment increased the nuclear translocation of Nrf2 (Fig 6D). However, the cytosolic Nrf2 levels remained unchanged or only slightly reduced. Meanwhile, we noticed that MCL treatment significantly induced total Nrf2 protein expression. We speculated that MCL-induced Nrf2 expression compensated for the decrease of cytosolic Nrf2. This may gave the explanation why the level of Nrf2 in cytosol remained almost unchanged. This finding indicated that MCL activated Nrf2 antioxidant protective mechanism in BV2 microglia.

### DMAMCL attenuated LPS-induced IL-6 expression in the cortex and hippocampus in mice

**Fig 7:**
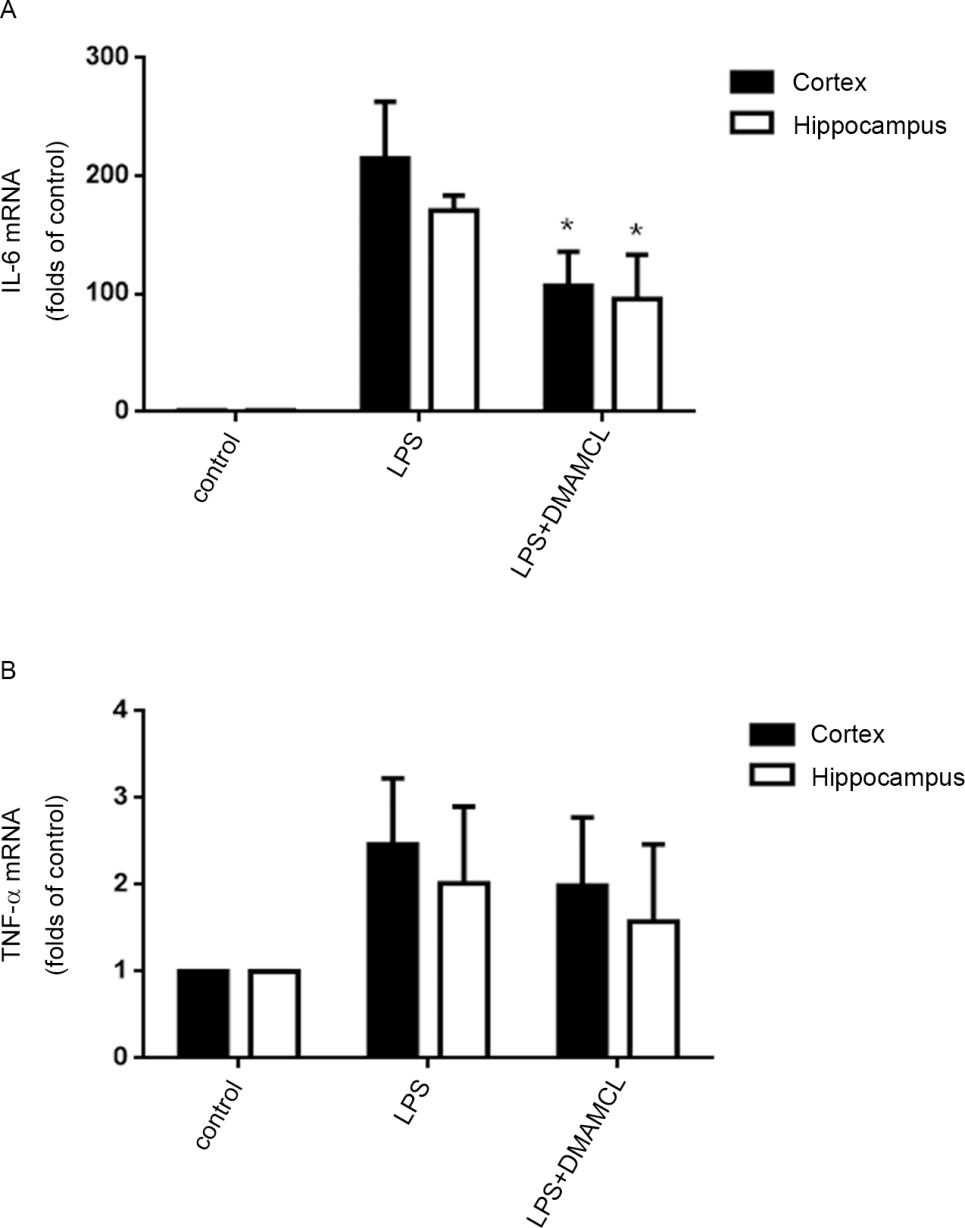
DMAMCL alleviated LPS-induced IL-6 expression in the cortex and hippocampus in mice. Mice were administered with 100 mg/kg DMAMCL by gavage for five consecutive days. On the fifth day, mice were challenged with saline or 0.33 mg/kg LPS i.p for 3 h. Then, the cortex and hippocampus were collected and total RNAs were extracted. IL-6 (A) and TNF-α (B) mRNA levels were determined by RT-PCR. Data were presented as means ± SD (n = 6). *p < 0.05 vs. LPS alone.

We further investigated the anti-inflammatory effect of MCL in LPS-induced neuroinflammation in Balb/c mice. To avoid the potential toxicity of DMSO, the solvent of MCL, to animals, DMAMCL, which is the water-soluble, dimethylamino Michael adduct of MCL, and can slowly release MCL as a metabolite in mouse plasma [18], was used in vivo studies. As shown in Fig 7A, LPS markedly increased IL-6 mRNA levels in the cortex and hippocampus compared to the untreated mice. Conversely, DMAMCL pretreatment significantly decreased the LPS-induced IL-6 mRNA expression in both brain regions. We also observed DMAMCL reduced LPS-stimulated the elevation of TNF-αmRNA levels in the cortex and hippocampus, but the difference did not reach significant level (Fig 7B). These results implied that MCL might suppress LPS-induced neuroinflammation in vivo.

## Discussion

MCL has been described to possess anti-inflammatory properties. For example, MCL can suppress LPS-stimulated cytokines production such as IL-6, TNF-α, MCP-1, et al, in Raw264.7 cells, primary macrophages, dendritic cells and human monocytes [24, 26]. However, whether MCL can inhibit neuroinflammation in microglia is unclear. LPS, a component of the outer membrane of gram-negative bacteria, is widely known as a potent stimulant that causes neuroinflammatory responses and brain damage [27, 28]. The BV2 cell is an immortalized murine microglial cell line and has been used frequently as a substitute for primary microglia [29]. In this study, we investigated the anti-inflammatory effects of MCL in LPS-stimulated BV2 cells and mice. Our results demonstrated that MCL could suppress LPS-stimulated multiple pro-inflammatory mediators expression including NO, COX-2, TNF-α, IL-1β and IL-6 both in BV2 cells and in mouse brain (Figs 2, 3 and 7).

We further explored the anti-inflammatory mechanism of MCL in LPS-activated BV2 cells. NF-κB has been known to play critical roles in microglial activation and is a predominant transcription factor in regulating pro-inflammatory mediators, such as iNOS, COX-2 and TNF-α [9, 30, 31]. In addition, MAPKs and PI3K/Akt signaling pathways are also engaged in modulating microglial inflammatory responses via up-regulation of NF-κB activity and subsequent cytokines expression [3, 10, 11]. Furthermore, redox-sensitive transcription factor Nrf2 plays an important role in cellular antioxidant defense [32]. LPS-induced microglia activation can be prevented by enhancing Nrf2 expression [33]. Moreover, phytochemical-induced activation of Nrf2 is reported to repress NF-κB activity [34]. HO-1 is one of the important genes in response to oxidative stress and functions to protect against various inflammatory diseases [35]. HO-1 and its product, carbon monoxide, can suppress the expression of pro-inflammatory mediators COX-2 and iNOS, thereby reducing COX-2-drived PGE_2_ and iNOS-derived NO production [36]. During oxidative conditions, Nrf2 translocates to the nucleus, binds to antioxidant response element, and regulates the transcription of antioxidant genes such as HO-1 [37]. Therefore, NF-κB, MAPKs, PI3K/Akt, and Nrf2/HO-1 signaling pathways are critical targets for anti-inflammatory response. In this study, we demonstrated that MCL could remarkably inhibit LPS-stimulated multiple inflammatory regulators’ activities including NF-κB, MAPKs such as JNK, p38, and ERK1/2, as well as Akt, simultaneously. Meanwhile, MCL could also promote Nrf2 expression and transcriptional activity to induce HO-1 expression (Figs 4–6). Although MCL can exert on multiple pathways, however, the direct targets of MCL are currently unknown and need to be further explored in the future.

Aberrantly activated microglia produce excessive amounts of various pro-inflammatory mediators, which can lead to neuroinflammation and neurodegenerative diseases [4–8]. Therefore, suppression of aberrant activation of microglia may have valuable therapeutic potential for the treatment of neuroinflammation-related neurodegenerative diseases. In this study, we showed that MCL strongly suppressed LPS-stimulated NO production and iNOS, COX-2 expressions, as well as various pro-inflammatory cytokines induction including TNF-α, IL-1β, and IL-6 both in BV2 cells and in mice. The mechanistic study suggested that the anti-inflammatory effects of MCL, on the one hand, partly depended on the inhibition of the activities of IκBα/NF-κB, MAPKs and Akt pathways, on the other hand, partially relied on the activating Nrf2/HO-1 anti-inflammation pathways. β-Lapachone (β-LAP), a natural naphthoquinone compound isolated from the lapacho tree (Tabebuia sp.), is reported to suppress LPS-induced neuroinflammation in BV2 cells and in mouse brain with similar pathways and mechanism to MCL [38]. However, β-LAP has low BBB permeability and only a minimal concentration of β-LAP penetrates into the brain, compared with other organs, under normal conditions. Therefore, the authors suggest two possible mechanisms of anti-neuroinflammatory effect of β-LAP on mouse brain: (1) β-LAP suppresses the peripheral inflammation induced by LPS and thus inhibits subsequent brain inflammation. (2) BBB permeability is compromised in systemic inflammatory conditions, hence the penetration of β -LAP into the brain might be enhanced [38]. In contrast, MCL can cross BBB and preferentially accumulates in the brain [17]. In addition, MCL is very stable in vitro and in vivo and no apparent side effects are observed after long-term treatment in vivo [18]. All this advantages and the wide anti-inflammation spectrum of MCL revealed in this study made it an ideal drug candidate to treat neuroinflammation-related neurodegenerative diseases.

## Materials and methods

### Reagents and antibodies

MCL and DMAMCL were generously provided by Accendatech Co., Ltd. (Tianjin, China) and were dissolved in DMSO (Sigma) and sterile water, respectively. LPS (Sigma) was dissolved in sterile water. Nitric oxide (NO) assay kit was purchased from Beyotime Institute of Biotechnology.

The following antibodies were used in this study: anti-iNOS, anti-phospho-ERK, anti-phospho-JNK, anti-p38, anti-phospho-Akt, anti-Akt, anti-p65, anti-phospho-IκBα, and anti-IκBα were from Cell Signaling Technology. Anti-COX-2 and anti-HO-1 were from Abcam. Anti-phospho-p38 was from Santa Cruz. Anti-Nrf2, anti-ERK, anti-c-Fos, anti-Histone H3, and anti-JNK were from Ruiying Biological. Anti-GAPDH and anti-β-tubulin were from Bioworld. Horseradish peroxidase conjugated secondary antibodies were from Macgene.

### Cell culture

BV2 cells were from Peking Union Medical College, Cell Bank (Beijing, China) and were cultured at 37 °C in 5% CO_2_ in DMEM (Macgene) in supplemented with 10% FBS (Hyclone).

### Measurement of cell viability

Cell viability was determined by CCK-8 cell counting kit (Vazyme). BV2 cells were plated into 96-well culture plates at a density of 1 × 10^4^/well in 100 μL volume and grown at 37 °C for 24 h. The culture medium was subsequently replaced by medium containing different concentrations of MCL (0, 1, 2, 5, and 10 μM). At the point of 20 h, 10 μL CCK-8 reagent was added to each well. After 4 h of incubation, the optical density of each well was determined at 450 nm using a microplate reader (Tecan).

### Measurement of NO production

BV2 cells were seeded in 6-well culture plates and pretreated with the indicated concentrations of MCL 1 h prior to stimulation with LPS (1 μg/ml) for 24 h. Cell supernatants were collected and assayed for NO production using Griess reagent. Briefly, cell supernatants were mixed with Griess reagent and then incubated at room temperature for 10 min. The optical density was detected under 540 nm and sodium nitrite was used as a standard curve.

### ELISA

BV2 cells were seeded in 6-well culture plates and pretreated with the indicated concentrations of MCL 1 h prior to stimulation with LPS (1 μg/ml) for 12 h. Then, cell supernatants were collected and centrifuged at 16,000 rpm for 10 min. The concentration of TNF-α and IL-6 were detected by specific ELISA kit (Neobioscience Technology Co., China) according to manufacturers’ instructions.

### Isolation of total RNA and RT-PCR

BV2 cells were pretreated with the indicated concentrations of MCL 1 h prior to stimulation with LPS (1 μg/ml) for 6 h and total RNA was extracted using RaPure Total RNA Kit (Magen) according to the manufacturer’s instruction. Reverse transcription was performed using RevertAid First Strand cDNA Synthesis Kit (Thermo Scientific). Real-time PCR analysis was performed using SYBR Select Master Mix (Life technologies) in conjunction with an ABI Prism 7500 Sequence Detection System with the expression of β-actin as the internal control. The data were analyzed using the ΔΔCT method. The primers for iNOS, COX-2, TNF-α, IL-1β, IL-6 and β-actin were described as below.

iNOS: F: 5’-GAAGAAAACCCCTTGTGCTG-3’, R: 5’-GTCGATGTCACATGCAGCTT-3’;

COX-2: F: 5’-GATGTTTGCATTCTTTGCCC-3’, R: 5’-TGAAGCCATGACCTTTCGCATTAGCATGG-3’;

TNF-α: F: 5’-GAAAAGCAAGCAGCCAACCA-3’, R: 5’-CGGATCATGCTTTCTGTGCTC-3’;

IL-1β: F: 5’-AATGACCTGTTCTTTGAAGTTGA-3’, R: 5’-TGATGTGCTGCTGCGAGATTTGAAG-3’;

IL-6: F: 5’-ACAAGTCGGAGGCTTAATTACACAT-3’, R: 5’-TTGCCATTGCACAACTCTTTTC-3’; and

β-actin: F: 5’-TCCTCCTGAGCGCAAGTACTCT-3’, R: 5’-GCTCAGTAACAGT CCGCCTAGAAs-3’

### Cytosolic and nuclear fractionation

Cytosolic and nuclear extracts were prepared using nuclear and cytoplasmic extraction kit (Applygen Technologies), according to the manufacturer’s instructions.

### Western blot analysis

Cells were washed twice with ice-cold 1× PBS, harvested, and lysed in RIPA buffer (Applygen Technologies) with phosphatase inhibitor tablet (Roche Diagnostics) and protease inhibitor (Cocktails, AMRESCO). Cell lysates were then centrifuged for 15 min at 13,000 × g at 4 °C and supernatants were collected and protein concentrations were determined by BCA Protein Assay Reagent (Pierce). Cell lysates (15–30 μg) were subjected to 8–12% SDS-polyacrylamide gel electrophoresis (SDS/PAGE) and transferred to nitrocellulose membranes (Millipore). For western blotting analysis, membranes were incubated with primary antibodies for overnight at 4°C followed by incubation with a secondary antibody for 1h at r.t. Then the signals were detected by enhanced chemiluminescence or fluorescence according to the manufacturer’s recommendation.

### Immunofluorescence analysis

In the NF-κB nuclear translocation assay, BV2 cells were seeded onto cover slips in 24-well culture plates and pretreated with MCL (10 μM) 1 h prior to stimulation with LPS (1 μg/ml) for 1 h. The cells were then fixed with 4% paraformaldehyde for 10 min, washed with PBS, and permeabilized with 0.5% Triton X-100 for 10 min at room temperature. Samples were blocked with 0.5% BSA for 1 h at room temperature and then incubated with the antibody to the NF-κB p65 overnight at 4 °C. The samples were washed with PBS and incubated with Alexa Fluor® 488-conjugated secondary antibody (Abcam) for 1 h at room temperature. Finally, the samples were washed again with PBS and were stained with DAPI (Sigma).

In the cell toxicity assay, BV2 cells were seeded onto cover slips in 24-well culture plates and treated with the indicated concentrations of MCL for 24 h. The cells were then fixed with 4% paraformaldehyde for 10 min, washed with PBS, and permeabilized with 0.5% Triton X-100 for 10 min at room temperature. Samples were incubated with the FITC-Phalloidin (yeasen) at room temperature for 30 min in the dark. Finally, the samples were washed with TBST and were stained with Hoechst33258 (yeasen). All immunofluorescence images were captured with a confocal laser scanning microscope (Olympus FV1000).

### Luciferase assays

Transfection of the NF-κB reporter vectors and Renilla vectors as loading control into BV2 cells was performed using Lipofectamine 2000 (Invitrogen) following the manufacturer’s instructions. Cells were plated onto 12-well plates and grown overnight. Then, cells were cotransfected with 1 μg of the reporter vectors along with 4 ng of Renilla vectors. After 24h, cell lysates were collected, and the luciferase activities against Renilla luciferase activities were measured with the double-luciferase assay system (Promega) following the manufacturer’s instructions. To determine the effect of MCL on LPS-induced NF-κB activity, cells were treated with 10 μM MCL in the absence or presence of LPS and incubated for 6 h prior to harvesting cells for luciferase assay. Renilla luciferase activity was used as an internal control. The relative luciferase activity was then calculated by normalizing firefly luciferase activity to Renilla luciferase activity.

### In vivo anti-inflammation effect assay

Balb/c mice (female, 3–4 month old) were divided into three groups (control group, LPS group, DMAMCL group, n = 6). DMAMCL (100 mg/kg) was first treated by intragastric administration for five consecutive days. On the fifth day, mice were also injected i.p with saline or Escherichia coli LPS (0.33 mg/kg). After 3 h, the mice were sacrificed and the brains were harvested. The gene expressions of TNF-α and IL-6 in the cortex and hippocampus were detected by real-time PCR assay.

### Statistical analysis

Data were presented as the means ± SD. The Student’s t-test was used to analyze statistical differences between groups. A two-tailed P-value of less than 0.05 was considered significant.

## Supporting information

S1 Fig The time lines which shown when the cells were treated with MCL and LPS and collected for any experiments. (TIF)
