# Supplementary figures and images for: Micheliolide suppresses LPS-induced neuroinflammatory responses
Source: PLoS One. 2017 Oct 17;12(10):e0186592. doi: 10.1371/journal.pone.0186592 (PMC5645131; doi:10.1371/journal.pone.0186592)

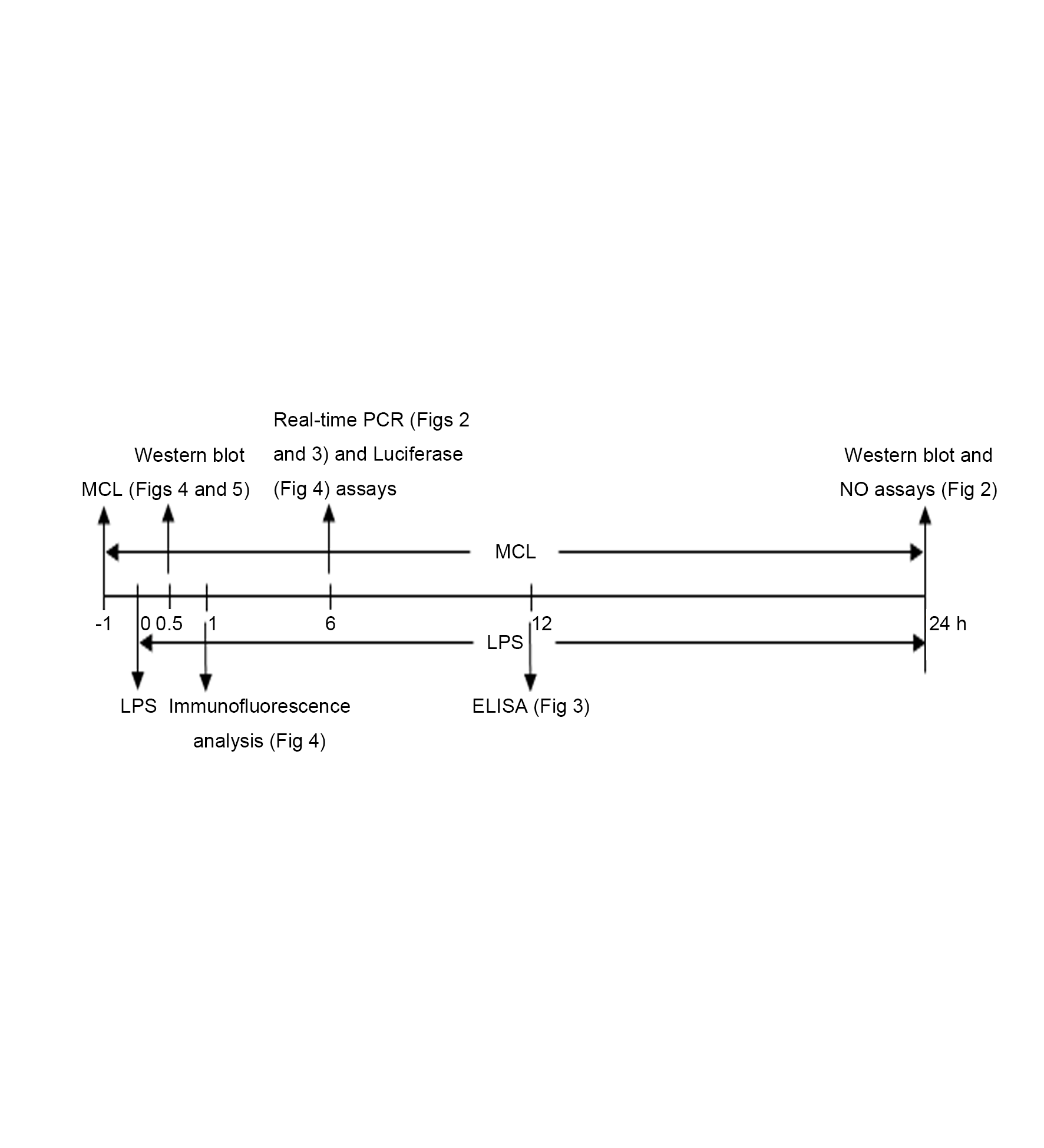

Supplement: S1 Fig — (TIF) [file pone.0186592.s001.tif]
